# Residual inflammation in psoriatic arthritis patients in stable minimal disease activity

**DOI:** 10.3389/fmed.2022.1096547

**Published:** 2022-12-20

**Authors:** Pierluigi Macchioni, Antonio Marchesoni, Giovanni Ciancio, Gilda Sandri, Alen Zabotti, Gentiana Vukatana, Luca Montaguti, Mariacristina Focherini, Marcello Govoni, Amelia Spinella, Nazzarena Malavolta, Francesca Zuliani, Marco Bruschi, Fabio Mascella, Carlo Salvarani

**Affiliations:** ^1^Rheumatology Unit, Arcispedale Santa Maria Nuova IRCCS, Reggio Emilia, Italy; ^2^Rheumatology, Humanitas San Pio X, Milan, Italy; ^3^Division of Rheumatology, University Hospital of Ferrara, Ferrara, Italy; ^4^Department of Rheumatology, Azienda Policlinico di Modena, University of Modena and Reggio Emilia, Modena, Italy; ^5^Department of Medical and Biological Sciences, Rheumatology Clinic, University of Udine, Udine, Italy; ^6^Rheumatology Unit, S. Orsola-Malpighi Polyclinic, Bologna, Italy; ^7^Rheumatology Unit, Azienda USL di Cesena, Cesena, Italy; ^8^Rheumatology Unit, Infermi Hospital, Rimini, Italy

**Keywords:** psoriatic arthritis, ultrasonography, minimal disease activity, synovitis, enthesitis

## Abstract

**Background:**

In psoriatic arthritis (PsA), low disease activity as defined by the Minimal Disease Activity (MDA) index is considered a good treatment target. However, as MDA is based only on clinical findings, it might not capture pauci-symptomatic inflammation. Sensitive imaging such as ultrasound (US) might disclose residual inflammatory signs in PsA patients in MDA.

**Methods:**

In this cross-sectional multicentre study, adult PsA patients on biologic treatment in MDA for at least 6 months were consecutively enrolled for a thorough clinical and US examination. Data collection included demographics, personal history, main patient's reported outcomes, clinical and US findings of joints, tendon sheaths, tendons, bursae, and entheses involvement. All centers performed the US investigation in B-mode and Power Doppler (PD)-mode using a similar US machine equipped with a 18–6 and 13–5 MHz multifrequency linear probe. Statistical analysis included comparisons between groups and correlation tests.

**Results:**

The 72 PsA patients enrolled in the study had a median duration of MDA of 12 (6–65) months. Overall, US examination revealed a low number of acute lesions. However, 54% of patients had at least one PD signal in the examined tissues. A joint or enthesis positive PD signal was found in about 19 and 24% of patients, respectively. Synovial hypertrophy, at least one acute entheseal lesions, and bursitis were the most common changes, detected in 41.7, 41.7 and 26% of patients, respectively.

**Conclusions:**

PsA patients in a stable state of MDA may still have residual inflammation in peripheral articular structures detectable by US examination.

## Introduction

According to the most recent recommendations, remission, or alternatively, minimal/low disease activity is the target to be achieved and maintained in patients with psoriatic arthritis (PsA) (1–3). A number of instruments are available to define disease state in these patients, all of them based on clinical findings, patient's reported outcomes, and, often, laboratory investigations (4). Currently, the Disease Activity index in Psoriatic Arthritis (DAPSA) (5) and the Minimal Disease Activity (MDA) (6) composite index are the most used assessment tools in clinical practice. Although these indices have a good accuracy in defining disease state, they might not detect asymptomatic underlying inflammation in entheses, joints, and other related structures. Ultrasound (US) studies have actually showed that PsA patients with a good disease control as measured by DAPSA or MDA may still have smoldering synovitis and enthesitis (7, 8). In addition, it is has been shown that a deeper assessment of disease activity by US may help to identify among patients in good disease control those who are at a greater risk of developing a flare (9).

In this study, we evaluated the presence of residual inflammation as detected by US in joints, entheses, tendon sheaths, and bursae of PsA patients with persistent good disease control.

## Patients and methods

### Study characteristics and patient selection

This was s a cross-sectional multicentre study conducted in the rheumatologic centers of seven Italian cities (Reggio Emilia, Modena, Cesena, Rimini, Ferrara, Udine, Bologna) with a specific expertise in spondyloarthritis (SpA) and US imaging. The study was approved by the local ethical committees of all participating centers according to the Italian current legislation on epidemiological studies.

### Study population

Adult patients (age 18–65 years) were consecutively enrolled by the participant centers if they had PsA according to the CASPAR classification criteria (10), if they were on therapy with a biological Disease Modifying Anti-Rheumatic Drug (bDMARD), alone or in association with a conventional synthetic (cs) DMARD, for at least 12 months, and if they were in a state of continuous MDA for at least 6 months. DMARD therapy could be associated with a stable dose (≤ 5 mg/day) of prednisone equivalent. Patients could be enrolled after giving their written informed consent, according to the Declaration of Helsinki. Patients participating in clinical trials or with any condition limiting their ability to comply with the study requirements were not eligible.

### Collection of clinical data

For each enrolled patients the following personal data were collected: demographics, anthropometry (including body mass index), life-style habits (including alcohol consumption, smoking and physical activity), social profile, concomitant diseases and previous and ongoing medications. The rheumatologic interview encompassed family history of arthritis, any inflammatory rheumatic condition, inflammatory bowel disease, uveitis, date of PsA onset and diagnosis, history of arthritis (pain and swelling in one or more joints), peripheral enthesitis (history of severe pain and limited function lasting at least 2 weeks in entheseal sites, particularly in the calcaneal areas), and dactylitis (history of a sausage digit), presence of inflammatory back pain according to the Assessment of SpondyloArthritis international Society (ASAS) criteria (11), of buttock pain and its distribution, and of anterior chest wall pain. The rheumatologic examination consisted of joint count (66 joints for swelling and 68 for tenderness), enthesitis evaluation, and dactylitis assessment (count and type of affected digit). The following entheses were examined for tenderness and swelling bilaterally: plantar fascia (PF) insertion on the calcaneus, Achilles tendon (AT), tibial tuberosity (TT), patellar tendon (PT), quadriceps tendon (QT), and common extensor tendon (CET) insertion on the lateral epicondyle of the humerus. Moreover, the Leeds Enthesitis Index (LEI) (12), and the Maastricht Ankylosing Spondylitis Enthesitis Score (MASES) (13) were calculated. The following data were also collected: Psoriasis Activity Severity Index (PASI) (14), Disability Index of the Health Assessment Questionnaire (DI-HAQ) (15), patient's pain (100 mm-visual analog scale, VAS), patient's global health (100 mm-VAS), physician's global evaluation of disease activity (100 mm-VAS), Bath Ankylosing Spondylitis Disease Activity Index (BASDAI) (16), Bath Ankylosing Spondylitis Function Index (BASFI) (17), and values of erythro-sedimentation rate (ESR) and C-reactive protein (CRP). DAPSA values and presence or absence of MDA were calculated using the appropriate data.

### US examination

US examination was performed in B-mode and PD mode using two US machines (ESAOTE MyLab70 or MyLabClass-C, Genoa, Italy) equipped with a 18–6 and 13–5 MHz multifrequency linear probe. The sonographers participating in this study had previously attended a training meeting that was preliminary to a US study in patients with PsA, psoriasis and fibromyalgia whose results have been recently published (18). The standardized protocol for US examination produced during this training was also adopted for the current study. All the operators were experienced in musculoskeletal ultrasonography and blinded to diagnosis and clinical findings. All of the images were recorded and the US evaluation was carried out the same day of the clinical examination. The following entheseal sites were scanned bilaterally in transverse and longitudinal planes: PF, AT, TT, PT, QT, and CET. In B-mode assessment, all the following abnormal findings were recorded: entheseal thickening measured at 2 mm proximal to the bony contour (abnormality definitions: quadriceps tendon >6.1 mm, proximal and distal patellar ligament >4 mm, Achilles tendon >5.29 mm, plantar fascia >4.4 mm), entheseal hypoechogenicity (defined as loss of normal fibrillar architecture), enthesophytes (defined as bony prominence at the end of the normal bone contour, seen in two perpendicular planes, with or without acoustic shadow), erosions (defined as a cortical break with a step down contour defect, seen in two perpendicular planes, at the insertion of the enthesis to the bone), and bursitis (defined as the presence of enlarged bursae at their anatomic sites as a well-circumscribed localized anechoic or hypoechoic area at the site of an anatomic bursa compressible by the transducer). These lesions were scored as 1 or 0 if present or absent. Entheseal thickening, entheseal hypoechogenicity, and bursitis were regarded as acute lesions. Bony erosions, calcifications, and enthesophytes were considered chronic lesions. Entheses were scored globally as 1 (presence of at least 1 lesion) and separately for acute and chronic involvement. Vascularisation was examined using PD-mode, standardized with a pulse repetition frequency of 750 Hz and a PD gain of 50 to 53 dB. Vascularisation was studied at the following areas: cortical bone insertion, body of tendon and bursa. The detection of vascularisation in any of these areas was considered abnormal. Enthesis US vascularity was classified into four distinctive patterns according to the number of vessels involved: 0 = none; 1 = 1–3 vessels; 2 = 4–5 vessels; 3≥5 vessels. The presence of PD ≥1 was considered indicative of an acute lesion. The patients were positioned as follows: knee entheses, patient in supine position and knee flexed at 70° for B-mode and in complete extension for PD-mode, AT and PF, patient in prone position with feet hanging off the examination bed, CET, patient sitting with arm laying on the examination bed and elbow flexed at 90°. US entheseal findings were scored according to the Madrid Sonography Enthesitis Index (MASEI) (19) and Glasgow Ultrasound Enthesitis Scoring System (GUESS) (20).

Wrists, metacarpo-phalangeal joints, knees and ankles were evaluated according to standardized methods: synovial hypertrophy, effusion and articular bone erosions were recorded as present or absent. The presence of intra-articular PD and synovial hypertrophy were recorded using a 4 grade scale according to Outcome Measures in Rheumatoid Arthritis Clinical Trial (OMERACT) criteria (0 = absent, 1 = low, 2 = medium, 3 = massive) (21). Tendon of the six carpal extensor tunnels, carpal and finger flexors at wrist, flexor and extensor of the fingers, flexor and extensor tendons of the feet were also evaluated at the dorsal and volar side of the wrist, dorsal and volar side of the hand and finger, ankle and dorsal foot areas for the presence of tendon sheath synovial hypertrophy and fluid distension as well as for the presence of tendon and tendon sheath PD signal according to European League Against Rheumatism (EULAR) recommendations (22).

### Statistical analysis

As this was an observational descriptive study, the sample size was not calculated. All patients meeting the inclusion criteria in the pre-defined time-frame of 1 year entered the study.

Continuous data were described as mean and standard deviation (mean + SD) or median and range, and categorical variables as absolute frequencies and percentages. Continuous variables were compared by using *t*-test or Mann-Whitney test when the distributions were skewed. Comparison of categorical variables was performed by using chi square or Fischer's exact test as appropriate. Correlations between variables were evaluated by Spearman's rho. Bonferroni correction was applied to the correlation results to counteract the multiple testing problem.

The comparison between patients with higher and lower clinical disease activity was performed using as cut-offs the DAPSA and BASDAI median values.

The level of significance was set at 0.05. Data were analyzed using the SPSS v.23 (IBM Statistics, Armonk, NY, USA).

## Results

Seventy-two patients with PsA in a state of MDA for at least 6 months were enrolled in this study; 40 of them with MDA for a 6–12 month period and 32 for more than 12 months. Their demographics, personal, and clinical data are reported in Tables 1, 2. All of the patients were bDMARD experienced and about one/third had been treated with csDMARDs. A the time of the US evaluation, all patients were taking bDMARDs, 24 of them in association with csDMARDs. A few of them were on low-dose oral corticosteroids but their US findings were not different from the others. They had been seen every about 3 months, a pre-established time-schedule for the patients taking bDMARDs in the participating centers. Mean and median values of the indices of articular and skin disease activity were all very low, confirming an overall good clinical control of disease. On average, patients had a long-standing state of well-controlled disease, as indicated by a median duration of MDA of 12 (6–65) months.

**Table 1:** Demographics and clinical characteristics of the 72 PsA patients included in the study.

**Mean age, year (SD)s**	**52.7 (13.9)**
Women, no (%)	29 (40.3)
Mean age at onset of psoriasis, years (SD)	32.1 (12.7)
Mean age at onset of PsA, years (SD)	39.3 (12.4)
Mean arthritis duration, years (SD)	13.6 (8.5)
Mean BMI (SD)	25.6 (3.57)
History of peripheral joint involvement, no (%)	68 (94.0)
History of peripheral entheseal involvement, no (%)	39 (49.2)
History of dactylitis, no (%)	44 (57.6)
History of nail involvement, no (%)	15 (24.7)
History of uveitis, no (%)	1 (1.4)
History of spine involvement, no (%)	12 (16.7)
bDMARDs line	
1st bDMARD, no (%)	18 (25)
2nd bDMARD, no (%)	44 (61)
3rd bDMARDs, no (%)	10 (14)
Mean duration of biological treatment, months (SD)	34.8 (23.5)
csDMARDs usage, no (%)	24 (33.1) MTX 20 (27.8) CsA 3 (4.2), LFN 1 (1.4)
Oral steroid usage, no (%)	8 (11)

bDMARDs, biological Disease Modifying Anti-Rheumatic Drugs; csDMARDSs, conventional synthetic DMARDs; MTX, methotrexate; CsA, Cyclosporine A; LEF, Leflunomide.

**Table 2:** Values of the indices of disease assessment in the 72 PsA patients included in the study.

	**Mean (SD)**	**Median (range)**
Duration of MDA, months	21.7 (23.1)	12 (6–65)
VAS pain, 0-100 mm	8.9 (13.9)	4.0 (0–7)
VAS general health. 0–100 mm	11.4 (17.2)	5.0 (0–7)
DI-HAQ	0.22 (0.30)	0.125 (0–1.5)
ESR, mm/1st h	10.6 (10.9)	8 (2–41)
CRP, mg/dl	0.20 (0.20)	0.12 (0–0.81)
PASI	0.43 (1.5)	0 (0–9)
Tender joint count	0.68 + 1.38	0 (0–7)
Swollen joint count	0.07 + 0.31	0 (0–2)
LEI	0.22 + 0.59	0 (0–3)
MASES	0.33 + 1.09	0 (0–7)
BASDAI	1.58 (1.51)	1.5 (0–3.1)
BASFI	1.07 (1.22)	0.67 (0–3.5)
DAPSA	2.57 (3.13)	1.6 (0.02–8.03)

MDA, Minimal Disease Activity; VAS, Visual Analog Scale; DI–HAQ, Disability Index of the Health Assessment Questionnaire; ESR, Erythro–sedimentation rate; CRP, C–Reactive Protein; PASI, Psoriasis Activity Severity Index; LEI, Leeds Enthesitis Index; MASES, Maastricht Ankylosing Spondyltis Enthesitis Score; BASDAI, Bath Ankylosing Spondylitis Disease Activity Index; BASFI, Bath Ankylosing Spondylitis Functional Index; DAPSA, Disease Activity index in PsA.

The results of the US evaluation are shown in Table 3. Overall, the mean number of joints, tendon sheaths, tendons, bursae, and entheses with signs of acute inflammation was very low. Nevertheless, at least a positive PD signal in the examined tissues was found in 39 patients (54.2%), at least one joint with a positive PD signal in 14 patients (19.4), and at least one entheses with a PD signal in 17 patients (23.6%). The mean and median values of PD entheseal and synovial scores were 1.49 + 0.61 and 1 (1–3), and 1.46 + 0.59 and 1 (1–3), respectively. Synovial hypertrophy, at least one acute lesion in the entheses, and bursa enlargement were the most common changes, detected in 30 (41.7%), 30 (41.7%) and 19 (26%) patients, respectively. Interestingly, at least one chronic lesion in the entheses was seen in 67 patients (93.1%).

**Table 3:** US abnormalities in the 72 PsA patients included in the study.

**Patients with at least one joint with effusion, no (%)**	42 (58.3 %)			
Joint with synovial effusion, mean (SD) and median (range)	1.32 + 1.63	2.26 + 1.54^*^	1.0 (0–6)	2.0 (1–6)^*^
Patients with at least one joint with synovial hypertrophy, no (%)	30 (41.7 %)			
Joint with synovial hypertrophy, mean (SD) and median (range)	0.71 + 1.0	1.70 + 0.87^*^	0 (0–4)	2 (1–4)^*^
Patients with at least one joint with positive PD signal, no (%)	14 (19.4 %)			
Joint with PD positive signal, mean (SD) and median (range)	0.44 + 1.19	2.29 + 1.82^*^	0 (0–8)	2 (1–8)^*^
Patients with at least one tendon sheath distension, no (%)	14 (19.4 %)			
Tendon with sheath distension, mean (SD) and median (range)	0.76 + 1.94	3.06 + 2.88^*^	0 (0–10)	2 (1–10)^*^
Patients with at least one tendon with PD signal, no (%)	5 (6.9 %)			
Tendon with PD signal, mean (SD) and median (range)	0.19 + 0.81	3.40 + 3.43^*^	0 (0–5)	2 (1–5)^*^
Patients with at least one enthesis with chronic lesions, no (%)	67 (93. 1%)			
Enthesis with chronic lesions, mean (SD) and median (range)	4.87 + 2.73	5.24 + 2.46^*^	5.0 (0–11)	5.0 (1–11)^*^
Patients with at least one enthesis with acute lesions, no (%)	30 (41.7 %)			
Enthesis with acute lesions, mean (SD) and median (range)	0.86 + 1.17	1.68 + 0.89^*^	0 (0–4)	2 (1–4)^*^
Patients with at least one enthesis with hypoechogenicity, no (%)	11 (15.3 %)			
Entheseal hypoechogenicity, mean (SD) and median (range)	0.15 + 0.36	1.91 + 0.83^*^	0 (0–1)	2 (1–3)^*^
Patients with at least one enthesis with PD signal, no (%)	17 (23.6 %)			
Entheseal PD, mean (SD) and median (range)	0.63 + 1.35	2.0 + 0.94^*^	0 (0–5)	2 (1–5)^*^
Patients with at least one bursa enlargement, no (%)	19 (26 %)			
Bursa enlargement, mean (SD) and median (range)	0.45 + 0.87	1.68 + 0.89^*^	0 (0–4)	(0–4)^*^
Total PD lesions, mean (SD) and median (range)	1.48 + 1.94	2.71 + 1.89^*^	1 (0–11)	3 (0–11)^*^
Patients with at least one PD lesion, no (%)	39 (54.2 %)			

* Mean and median values of the patients who had US lesions.

The comparisons between patients with a DAPSA score greater or lower than 1.6 and a BASDAI values greater or lower than 1.5 are shown in Table 4. The only significant differences were the number of patients with synovial PD signal (higher in the >1.5-DAPSA group) and the number of patients with joint effusion (higher in >1.6-BASDAI group).

**Table 4:** Number of patients with acute lesions according to DAPSA and BASDAI scores.

	**DAPSA <1.6 (36 pts)**	**DAPSA > 1.6 (36 pts)**	**p OR (95%CI)**	**BASDAI <1.5 (35 pts)**	**BASDAI >1.5 (37 pts)**	**p OR (95%CI)**
Patients with at least one joint with positive PD signal, no (%)	3 (8.3%)	11 (30.6%)	0.035 0.207 (0.052–0.82)	10 (28.6%)	4 (10.8%)	0.076 0.303 (0.085–1.08)
Patients with at least one tendon with PD signal, no (%)	0 (0%)	5 (13%)	0.054 0.86 (0.75–0.98)	3 (8.6%)	2 (5.4%)	0.770 1.41 (0.22–9.01)
Patients with at least one enthesis with acute lesions, no (%)	14 (42%)	16 (52%)	0.462 0.691 (0.26–1.85)	13 (37.1%)	10 (27.0%)	0.358 0.627 (0.23–1.70)
Patients with at least one joint with effusion, no (%)	18 (50%)	24 (66.7%)	0.151 0.50 (0.16–1.30)	16 (45.7%)	26 (70.3%)	0.036 2.81 (1.06–7.40)

The correlation tests revealed the following weak but significant associations: number of joints with effusion at US with HAQ-DI (coefficient 0.368, *p* = 0.003), with BASDAI (coefficient 0.364, *p* = 0.002), with VAS global (coefficient 0.279, *p* = 0.018) (not significant after Bonferroni correction), and with BASFI (coefficient 0.271, *p* = 0.023) (not significant after Bonferroni correction). A negative correlation was found between months of MDA duration and number of joints with effusion (coefficient −0.275, *p* = 0.01), number of articular or periarticular structures with PD signal (coefficient −0.343, *p* = 0.006), and number of enthesis with hypoecogenicity (coefficient −0.274, *p* = 0.02) but none of these correlations remained significant after Bonferroni correction.

Finally, only 2 (8.2%) of the 24 patients taking csDMARDs had positive joint PD signal, as opposed to 12 patients (25%) not taking them (*p* = 0.092) and the comparison according to the duration of MDA revealed a significantly higher number of patients with at least one US-PD lesions in the ≤ 12- than in the >12-month group, 22 (55%) and 13 (41%), respectively (*p* = 0.024).

## Discussion

The results of this study suggest that patients with PsA with a persistent good clinical control of disease may still have joint and/or entheseal inflammation as revealed by US imaging. A positive PD signal in at least one joint and/or enthesis was found in about 19 and 24% of patients, respectively. As the mean number of joints and entheses with US signs of acute inflammation was very low, in most patients this involvement was limited to one or few sites. US findings of “active inflammation” in joints and entheses can also be found in healthy subjects. In a recent study, US acute lesions in the entheses were seen in up to almost 35% of healthy subjects but a positive PD signal was detected in < 10% of them (23). In another study, US acute changes were frequently seen in joints of healthy subjects, with joint effusion, synovial hypertrophy, and positive PD found in 52, 13, and 5% of these subjects, respectively (24). In our study a PD signal in the entheses was found in 24% of patients and the prevalence of US acute lesions in the joint was relatively high, especially for PD signal (19%). In addition, bursa enlargements and tendon sheath distensions were seen in a proportion of patients (19 and 26%, respectively) too high to be considered normal findings (25). Altogether, these data indicate that the acute US lesions that we found in joints and entheses of a population of PsA patients with long-standing MDA were probably due to residual disease. They also suggest that PD signal in both joints and entheses is the US lesion more suggestive of true disease activity. An indirect indication that US findings of acute inflammation might be due to the articular disease was the significant correlation between number of joint with effusion at US and several patient reported outcomes.

The definition of MDA (6) allows for the presence of mild clinical and laboratory inflammation. In our study, however, all the indices of entheseal and joint assessment, all of the patient's reported outcome values, and DAPSA scores were very low, indicating that there were no or very little sign of clinically active disease. Therefore, the US findings of synovitis and enthesitis were largely found in otherwise silent sites. This result was not surprising, as in PsA sonography examination of joints and entheses has been shown to be more sensitive than clinical assessment and the correlation between clinical and sonographic remission was, at best, moderate (7, 8, 26, 27).

Whether the presence of smoldering joint and entheseal inflammation by US may be of clinical relevance should be investigated by longitudinal prospective studies. One study on PsA patients in MDA, showed that US-detected synovitis was a strong predictor of disease flare (9). Similar data have been reported for rheumatoid arthritis (RA) (28). Therefore, PsA patients in stable MDA but with underlying signs of US inflammation might be at greater risk of disease flare. These patients might need a tighter follow-up and might not be candidate for therapy tapering. However, the benefit of using sonographic remission as a target in PsA in a treat-to-target strategy should be carefully evaluated. In fact, it was shown that in RA targeting remission with US was not better than using clinical instruments of disease assessment (29, 30).

Another interesting result yielded by this study was that the patients with a longer period of MDA had less US acute lesions than those with a shorter time of MDA. This finding might suggests that a long-lasting disease control may lead to a deeper state of remission. This possibility was also corroborate by the negative correlation between duration of MDA and number of joint and enthesis US acute changes.

The study has some limitations. First, as the sample size was not pre-calculated, the number of evaluated patients might not have been adequate for a fully powered statistical analysis. Second, all patients were in stable good disease control and the number of findings revealed by the US examination was relatively low. As a result, some differences might not have achieved the level of significance. Finally, the cross-sectional design of the study, did not allow to understand the prospective usefulness of the US findings. The main results of our study, however, should not be impaired by these limitations.

In conclusion, this study showed that in a group of PsA patients in a state of long-standing MDA, US examination revealed the presence of residual inflammation in joints, entheses, tendon sheaths and bursae. Therefore, sonography might help to better define the state of disease activity in the individual PsA patient in persistent clinical MDA.

## Data availability statement

The raw data supporting the conclusions of this article will be made available by the authors, without undue reservation.

## Ethics statement

The studies involving human participants were reviewed and approved by Ethics Committee of the Santa Maria Nuova Hospital, Reggio Emilia, Italy. The patients/participants provided their written informed consent to participate in this study.

## Author contributions

PM: study design, patient enrollement, and data analysis. AM: data interpretations and drafting of the manuscript. GC, GS, AZ, GV, LM, MF, MG, AS, NM, FZ, MB, and FM: patient enrollment. CS: work conception and coordination. All authors contributed to the article and approved the submitted version.
